# Quality of YouTube Videos on Laparoscopic Cholecystectomy for Patient Education

**DOI:** 10.1155/2021/2462832

**Published:** 2021-09-15

**Authors:** Joseph N. Hewitt, Joshua G. Kovoor, Christopher D. Ovenden, Gayatri P. Asokan

**Affiliations:** ^1^University of Adelaide, Discipline of Surgery, The Queen Elizabeth Hospital, Adelaide, SA, Australia; ^2^University of Adelaide, Discipline of Surgery, Women's and Children's Hospital, Adelaide, SA, Australia

## Abstract

**Background:**

Surgical patients frequently seek information from digital sources, particularly before common operations such as laparoscopic cholecystectomy (LC). YouTube provides a large amount of free educational content; however, it lacks regulation or peer review. To inform patient education, we evaluated the quality of YouTube videos on LC.

**Methods:**

We searched YouTube with the phrase “laparoscopic cholecystectomy.” Two authors independently rated quality of the first 50 videos retrieved using the JAMA, Health on the Net (HON), and DISCERN scoring systems. Data collected for each video included total views, time since upload, video length, total comments, and percentage positivity (proportion of likes relative to total likes plus dislikes). Interobserver reliability was assessed using an intraclass correlation coefficient (ICC). Association between quality and video characteristics was tested.

**Results:**

Mean video quality scores were poor, scoring 1.9/4 for JAMA, 2.0/5.0 for DISCERN, and 4.9/8.0 for HON. There was good interobserver reliability with an ICC of 0.78, 0.81, and 0.74, respectively. Median number of views was 21,789 (IQR 3000–61,690). Videos were mostly published by private corporations. No video characteristic demonstrated significant association with video quality.

**Conclusion:**

YouTube videos for LC are of low quality and insufficient for patient education. Treating surgeons should advise of the website's limitations and direct patients to trusted sources of information.

## 1. Introduction

Use of the Internet by patients seeking health information is ubiquitous. Patients turn to information on the Internet both before seeking help from a professional and also after or as a second opinion [1]. In particular, the YouTube video sharing platform may be used by patients to learn about surgical procedures [2]. Laparoscopic cholecystectomy is one of the most common procedures performed by general surgeons [3]. Effective methods of delivering patient education prior to these operations is required to optimise clinical care, and YouTube videos represent a novel way this can be achieved.

In the midst of the coronavirus disease 2019 (COVID-19) pandemic, social and physical distancing recommendations there have been rapidly increase in the use of telehealth solutions [4]. In this context, it is even more important to be aware of the nature of online health information that patients might turn to, to supplement or supplant face to face contact with their doctor.

Previous studies have characterised the quality of YouTube videos for patient education prior to surgery and found them to be poor [5, 6]. A literature search found studies evaluating YouTube videos of cholecystectomy for use as education for surgeons in training [7, 8] and to educate patients on “gallstone disease,” reaching similar conclusions [9]. To our knowledge, no study has evaluated YouTube videos on cholecystectomy from a patient information perspective using validated scoring tools. Thus, to inform surgical patient education, we aimed to evaluate the quality of information available to patients on YouTube regarding laparoscopic cholecystectomy and identify video characteristics associated with increased quality.

## 2. Methods

We adapted methods outlined by Ovenden and Brooks [6]. A YouTube search was conducted for “laparoscopic cholecystectomy” on 24 November 2020, and the first 50 video search results were included in the study. The search was conducted by one author (JNH) in English, using a web browser with cookies cleared to prevent search results influenced by previous web activity, with no additional filters applied. The results of the search were then distributed to the other authors for data extraction and analysis.

For each video, we extracted data for search results, date of publication, length, number of views, number of comments, and number of ‘likes' and ‘dislikes' and assigned an authorship category. We defined ‘positivity' as the ratio of video ‘likes' to the sum of ‘likes' and ‘dislikes' expressed as a percentage.

Two authors (JNH and JGK) independently watched and scored each video against three previously validated scoring systems: the Journal of the American Medical Association (JAMA) [10], Health on the Net (HON) [11], and DISCERN [12]. The mean of the two authors' scores was used as a consensus score in further analyses. JAMA assesses authorship, attribution, currency, and disclosure, each scoring zero or one points for a total of four. HON scores authoritativeness, complementarity, privacy, attribution, justifiability, transparency, financial disclosure, and advertising policy, again each scoring zero or one for a total of eight possible points. DISCERN scores fifteen criteria between one and five points along with one criteria of overall quality and assigns the mean of all these as the final score. The aggregate of subscores for each domain was added together for all videos to ascertain which areas were most lacking in videos. The scoring systems are attached as appendices.

Prior to video scoring, the authors had a discussion regarding what would be considered the ideal video and how points would be awarded. The scoring process was then undertaken independently.

We tested interobserver reliability with an intraclass correlation (ICC) analysis and then used the mean score from both observers for further analysis. Linear regression or Student's *t*-tests were used to test for relationships between scores assigned and video characteristics. All statistical analysis was performed with IBM SPSS ver. 27 (IMB Corp., NY, USA).

## 3. Results

Of the 50 videos analysed, 26 (52%) were authored by surgeons, 10 (20%) by healthcare or education companies, and 7 (14%) by hospitals or universities, and in 7 cases (14%), video authorship fell outside these categories or was unclear. Five videos were authored by the same surgeon and 2 videos by the same healthcare or education company. Extracted data regarding video characteristics are outlined in Table 1.

There was good interobserver reliability for all three scoring systems with ICC values 0.78, 0.81, and 0.74 for JAMA, DISCERN, and HON scores, respectively. The mean (+/− SD) video quality as measured by each score was 1.9/4.0 (±0.7), 2.0/5.0 (±0.6), and 4.9/8.0 (±0.9) for JAMA, DISCERN, and HON, respectively. There was a statistically significant correlation between video quality as measured by each score and as measured by both other scores. This is demonstrated in Table 2.

There was no significant association between any video characteristics and video quality as measured by any of the three scoring systems. There was a positive correlation between the number of views and number of comments (*p* < 0.001, Pearson's correlation). There was a correlation between video rank in search results and number of views (*p* < 0.001, Spearman's correlation) and comments (*p*=0.002, Spearman's correlation) but no correlation between video rank and quality as measured by each score.

For the JAMA score, the best scored attribute across all videos was authorship with 67% of videos achieving a point. The lowest scoring attribute was disclosure with only 7% of videos gaining a point. For HON, the highest and lowest scoring categories were authoritativeness (74%) and complementarity (15%). For DISCERN, relevance (62%) and discussion of risks (24%) were the highest and lowest scoring categories.

## 4. Discussion

The quality of YouTube videos on laparoscopic cholecystectomy for patient education is suboptimal when assessed by the JAMA, DISCERN, and HON scoring systems. There was good intraclass correlation between scorers for each of these systems. This video series contained a higher proportion of videos produced by surgeons and, significantly, no patient testimonial videos. This is in contrast to the series of videos Ovenden and Brooks obtained on anterior cervical discectomy which included nearly 50% patient testimonials [6]. The reason for this difference is unclear but may be a product of the link between psychosocial stress and spinal disorders [13]. This link is not evident for laparoscopic cholecystectomy.

There was no correlation in our series between high video quality and the number of views. While YouTube's algorithm for search recommendations remains an unknown black box [14], it is not possible to know how videos are recommended to users, but our analysis suggests the algorithm does not select for videos of high quality. We did observe a correlation between the number of views and a high ranking in search results which is likely a product of the high-ranking videos being more visible and accessible. There are likely other factors which we did not analyse which predicts a video success in the search algorithm and, hence, the number of views.

The management of surgical systems has been modified considerably at many points during the current COVID-19 pandemic [15]. Recent evidence suggests that some of these changes may need to be enduring to ensure surgical safety as COVID-19 continues to spread [16]. Throughout this adaptation process, the quality of the patient education process must be maintained, particularly for high-volume procedures such as laparoscopic cholecystectomy. Our study investigated the use of YouTube videos on these procedures and found that quality was inadequate to advocate for implementation as a primary mechanism of preoperative patient education. It is of some concern that despite the low quality of the videos, the percentage positivity was generally high, indicating that viewers approved of potentially erroneous and unreliable information. However, the accessibility of this information and high degree of involvement of surgeons in the creation of these videos mean that potential utility may exist as a supplementary form of patient education alongside standard in-person or telehealth consultation.

Patient education before operations such as laparoscopic cholecystectomy typically consists of pamphlets and verbal information provided by the surgeon and nurses during a preadmission consultation [17]. While our study found that YouTube videos are not of high enough quality to replace this educational infrastructure, they may have utility as a form of supplemental information supplementation that is free and easily accessible. However, if surgeons are to direct patients to YouTube videos at any point of preoperative care, individual patient factors must be considered. Within the retrieved videos that were analysed, we found multiple different forms of information provision. These included raw intraoperative footage with or without surgeon narration, animations or cartoons explaining intraoperative approaches and perioperative care, and a brief interview with a specialist surgeon. Regarding videos including intraoperative footage, some patients may be intrigued by seeing real-life anatomy while others may be perturbed. Regarding animations or cartoons, some patients may be visual learners and benefit from idealised diagrammatic representations of anatomy and procedures, while others may view the medium as overly generic. Regarding interviews with specialist surgeons, some may see value in receiving a renowned expert's opinion, while others may not value it if it is not individualised to their specific clinical picture. As with any other aspect of patient-centred surgical care, direction to YouTube videos as a supplemental form of preoperative education should be individualised to each respective patient. It must be noted that discussion of the risks inherent to a surgical procedure scored poorly across videos overall and this is a crucial aspect of the informed consent process. The lack of disclosure of sources of funding for videos and possible conflicts of interest was also apparent across aggregate scores. These are two areas which patients must, therefore, be wary of when accessing such videos and which surgeons should be mindful to include, were they to produce a video.

Approaches to surgical intervention have evolved rapidly over time [18]. Although the consensus approach to laparoscopic cholecystectomy has not changed significantly in recent years [19], this trend may not continue as novel data are published in the literature. In our study, the median video age was 4.3 years, suggesting there is scope to provide new videos of a high quality which more accurately represent contemporaneous practice. Alternatively, if referring patients to YouTube for supplementary preoperative education, surgeons can advise the implementation of temporal restrictions on searches in line with major developments in the field.

This study has multiple limitations. Primarily, we only analysed the first 50 search results obtained from YouTube. However, this amount was selected pragmatically, as it is within the scope of retrieved videos which the usual searcher would be most likely to view. YouTube was the only platform used in this study, potentially limiting the generalisability of our results to other online health information sources. “Laparoscopic cholecystectomy” was the only search term, and there may be higher-quality videos for patient education available by searching “gallbladder operation,” for example. Five of the 50 videos were posted by the same surgeon and 2 by the same company, and this may further limit the generalisability of our results. This is a limitation inherent in YouTube's search algorithm, however, and these same videos could be presented to a patient who made the same search. There will always be an inherent level of subjectivity to qualitative assessments of these forms of videos; however, we sought to mitigate this limitation and ensure reliability of results through the use of three separate validated scoring methods. Within the scoring tools, certain items required knowledge of the intervention, alternative treatment approaches, and effectiveness within overall shared decision making between the patient and clinician. At the time of this study, the two authors who scored the videos were a surgical resident medical officer and final-year medical student, respectively, and, thus, did not have a specialist level of knowledge of laparoscopic cholecystectomy. To mitigate potential bias, we selected scoring tools that have been validated and do not require specialist knowledge or help to be used [12].

Surgeons should be aware that the quality of information their patients may have accessed regarding laparoscopic cholecystectomy online is suboptimal. This is even more important when, with the uptake of telehealth experienced along with the COVID-19 pandemic and the rise of digital health, patients may turn increasingly to sources of information other than their treating surgeon.

## Figures and Tables

**Table 1:** Video characteristics.

Median views	21,789 (IQR 3000–61,690)
Median age (months)	52.6 (IQR 23.1–91.6)
Median length (mm:ss)	10 : 09 (IQR 04 : 40 –17 : 16)
Median positivity	93% (IQR 90 –96%)
Median comments	14 (IQR 2–31)

**Table 2:** Correlation between scores measuring video quality (Pearson's correlation).

		JAMA	Discern	Hon
JAMA	Pearson correlation		0.364	0.775
	Significance		0.009	<0.001
Discern	Pearson correlation	0.364		0.523
	Significance	0.009		<0.001
Hon	Pearson correlation	0.775	0.523	
	Significance	<0.001	<0.001	

**Table 3:** Journal of the American Medical Association (JAMA).

Component	Score
Authorship: authors and contributors, their affiliations, and relevant credentials should be provided	0-1
Attribution: references and sources for all content should be clearly listed	0-1
Disclosure: website ownership should be prominently and fully disclosed, as should any sponsorship, advertising, or funding arrangements	0-1
Currency: dates that content was posted and updated should be indicated	0-1
Total	**0–4**

**Table 4:** Health on the Net (HON).

Component	Score
Authority: details of the editorial team are clearly stated	0-1
Complementarity: clear mention of site boundaries that do not replace the relationship between the physician and patient	0-1
Confidentiality: declaration explaining all legal requirements concerning the confidentiality of personal data	0-1
Attribution: the site has a date of last update, and sources are given	0-1
Justifiability: information is complete and provided in an objective, balanced, and transparent manner	0-1
Transparency: the site is easy to use	0-1
Financial disclosure: all sources of funding are identified and transparent	0-1
Advertisement policy: all advertisements should be identified and differentiated from content	0-1
Total	**0-8**

Adapted from https://www.hon.ch/HONcode/Visitor/visitor.html.

**Table 5:** Discern.

Component	Score
Are the aims clear?	1–5
Does it achieve its aims?	1–5
Is it relevant?	1–5
Is it clear what sources were used to compile the publication?	1–5
Is it clear when the information used or reported in the publication was produced?	1–5
Is it balanced and unbiased?	1–5
Does it provide details of additional sources of support and information?	1–5
Does it refer to areas of uncertainty?	1–5
Does it describe how each treatment works?	1–5
Does it describe the benefits of each treatment?	1–5
Does it describe the risks of each treatment?	1–5
Does it describe what would happen if no treatment is used?	1–5
Does it describe how the treatment choices affect the overall quality of life?	1–5
Is it clear there may be more than one possible treatment choices?	1–5
Does it provide support for shared decision making?	1–5
Rate the publication overall	1–5
Average	**1–5**

Adapted from https://www.discern.org.uk/discern_instrument.php.

## Data Availability

The data used to support the findings of this study are available on request to the corresponding author.

## Appendix

### A. Scoring Systems

Table 3 was adapted from Silberg WM, Lundberg G, Musacchio RA. Assessing, controlling, and assuring the quality of medical information on the Internet: caveat lector et viewor: let the reader and viewer beware. JAMA. 1997; 277:1244–1245.  Table 4 was adapted from www.hon.ch/HONcode/Visitor/visitor.html.  Table 5 was adapted from www.discern.org.uk/discern_instrument.php.
